# *De Novo* Structural Variations of *Escherichia coli* Detected by Nanopore Long-Read Sequencing

**DOI:** 10.1093/gbe/evad106

**Published:** 2023-06-09

**Authors:** Xia Zhou, Jiao Pan, Yaohai Wang, Michael Lynch, Hongan Long, Yu Zhang

**Affiliations:** Institute of Evolution and Marine Biodiversity, KLMME, Ocean University of China, Qingdao, Shandong Province, China; Institute of Evolution and Marine Biodiversity, KLMME, Ocean University of China, Qingdao, Shandong Province, China; Institute of Evolution and Marine Biodiversity, KLMME, Ocean University of China, Qingdao, Shandong Province, China; Biodesign Center for Mechanisms of Evolution, Arizona State University, Tempe, Arizona, USA; Institute of Evolution and Marine Biodiversity, KLMME, Ocean University of China, Qingdao, Shandong Province, China; Institute of Evolution and Marine Biodiversity, KLMME, Ocean University of China, Qingdao, Shandong Province, China; School of Mathematics Science, Ocean University of China, Qingdao, Shandong Province, China

**Keywords:** mutation accumulation, structural variations, mutation distribution, long-read sequencing

## Abstract

Spontaneous mutations power evolution, whereas large-scale structural variations (SVs) remain poorly studied, primarily because of the lack of long-read sequencing techniques and powerful analytical tools. Here, we explore the SVs of *Escherichia coli* by running 67 wild-type (WT) and 37 mismatch repair (MMR)–deficient (Δ*mutS*) mutation accumulation lines, each experiencing more than 4,000 cell divisions, by applying Nanopore long-read sequencing and Illumina PE150 sequencing and verifying the results by Sanger sequencing. In addition to precisely repeating previous mutation rates of base-pair substitutions and insertion and deletion (indel) mutation rates, we do find significant improvement in insertion and deletion detection using long-read sequencing. The long-read sequencing and corresponding software can particularly detect bacterial SVs in both simulated and real data sets with high accuracy. These lead to SV rates of 2.77 × 10^−4^ (WT) and 5.26 × 10^−4^ (MMR-deficient) per cell division per genome, which is comparable with previous reports. This study provides the SV rates of *E. coli* by applying long-read sequencing and SV detection programs, revealing a broader and more accurate picture of spontaneous mutations in bacteria.

**Significance Statement**

The complexity of eukaryotic and prokaryotic genomes raises challenges for detecting structural variations (SVs) with high-throughput sequencing data. Here, we compared SV detection results based on short- and long-read sequencing combined with multiple analysis callers, identifying the most suitable strategies for different SVs for simulated and real data of *Escherichia coli*. Our results provide reliable SV detection procedures for future research on bacterial mutations.

## Introduction

Spontaneous mutations occur in all living organisms and are the primary source of genetic variation. Common types of mutations are base-pair substitutions (BPSs), small insertions and deletions (indels), and large-scale structural variations (SVs). Most previous studies have focused primarily on BPSs and small indels due to sequencing technology limitations (Lee et al. 2012; Lynch et al. 2016; Long et al. 2018b; Pan et al. 2022). Although neglected or unresolved, early studies have found that many human diseases are associated with SVs. For example, duplication fragments of human chromosome 17p lead to Charcot–Marie–Tooth disease type 1A, and large homozygous deletions of the 2p13 region result in juvenile nephronophthisis (Lupski et al. 1991; Konrad et al. 1996). SVs also play essential roles in genome evolution: some beneficial SVs may help organisms adapt to their environments, and some copy number variant–dominated SVs are positively selected with higher frequencies (Emerson et al. 2008; Iskow et al. 2012; Kondrashov 2012). Differences in large-effect SVs of genes controlling specific traits at the population level imply that SVs may be associated with the formation of new species (Chan et al. 2010). Because most bacterial genomes are haploid, the fitness effects of SVs in bacteria are even more significant than those in humans. SVs have a profound impact on the evolution of bacteria, particularly for many pathogenic species, where the pathogenicity or new virulence phenotypes are associated with SV-carrying critical genes that are frequently caused by transposition or recombination events (Lieberman et al. 2011; Damkiær et al. 2013; Lee et al. 2016).

Previous studies have detected SVs mostly by using short paired-end reads (Ye et al. 2009; Iqbal et al. 2012; Rausch et al. 2012; Barrick et al. 2014; Deatherage and Barrick 2014; Fan et al. 2014; Layer et al. 2014; Chen et al. 2016; Lee et al. 2016; Tian et al. 2018). Such strategy has played a key role in the identification of SVs, revealing their diversity in individuals to population (Ma et al. 2021; Zhao et al. 2021a; Chen et al. 2022). Based on such analytical strategies, *E. coli* insertion sequence (IS) elements were reported to have an insertion rate of 3.5 × 10^−4^ and a recombination rate of 4.5 × 10^−5^ per genome per generation, and the transposition rate in *E. coli* measured by other methods was about 10^−5^ (Sousa et al. 2013; Lee et al. 2016). However, the accuracy of such explorations may be affected by the inherent defects of short-read sequencing (Putze et al. 2009; Lee et al. 2014; Mahmoud et al. 2019). In contrast, the combination of long-read sequencing and more advanced bioinformatics tools can provide unique anchors in the repeat regions of the reference genome and achieve better results for identifying breakpoints and more types of SVs (Cretu Stancu et al. 2017; Mahmoud et al. 2019). Such strategy has been greatly optimized for identifying SVs in complex and nested sequences or low-depth sequencing data (Sedlazeck et al. 2018; Tham et al. 2020). Consequently, long-read sequencing provides a more complete and precise view of *de novo* spontaneous mutations at all scales, although such trials are rarely performed.

Mutation accumulation (MA) combined with whole-genome sequencing is the most classical strategy for determining the rate and spectrum of spontaneous mutations (Foster 2006; Lee et al. 2012). Single-individual transfers repeatedly bottleneck large sets of parallel lines, so that genetic drift dominates selection, and even deleterious mutations can be accumulated, eventually providing nearly unbiased mutational features. MA of DNA mismatch repair (MMR) defective strains can further provide an accurate picture of mutations before the specific repairing of MMR (Iyer et al. 2006; Lee et al. 2012; Long et al. 2016; Long et al. 2018a). In this study, we tested and identified better strategies using in silico simulation, MA of wild-type (WT) and MMR-deficient *E. coli* K-12 MG1655, and Nanopore long-read and Illumina PE150 sequencing for analyzing bacterial SVs.

## Results

To detect SVs in the *E. coli* K-12 MG1655 genome, we accumulated *de novo* mutations by daily single-colony streaking 80 WT MA lines and 40 MMR-defective (Δ*mutS*) lines from 1 WT ancestor cell and 1 Δ*mutS* ancestor cell, respectively. Eventually, 67 WT and 37 Δ*mutS* MA lines were used for the final analysis after removing low-coverage, cross-contaminated lines or those with mutations falling in other repair systems (supplementary table S1, Supplementary Material online). Each WT MA line experienced about 4,480 cell divisions and was sequenced to a mean depth of coverage 99× (standard error, SE: 5.56) and 4,320 cell divisions and 123× (SE: 9.34) for the Δ*mutS* MA lines. More than 99% of the genomes of all the MA lines were covered with high-quality reads (supplementary tables S2 and S3, Supplementary Material online). We also performed Nanopore long-read sequencing on 19 WT and 18 Δ*mutS* MA lines as well as their ancestors (1 Δ*mutS* line was removed due to 3 mutations in the repair gene *mutT*) with ∼1 Gbp to 3 Gbp for each line (supplementary table S4, Supplementary Material online). The features of BPSs and small indels are highly consistent with previous studies, confirming the high repeatability of the *E. coli* mutation–accumulation experiments (supplementary file S1, figs. S1 and S2, and tables S2, S3, and S5–S13, Supplementary Material online).

### Evaluating the SV Detection Pipelines with Simulated Data

**Fig. 1.**
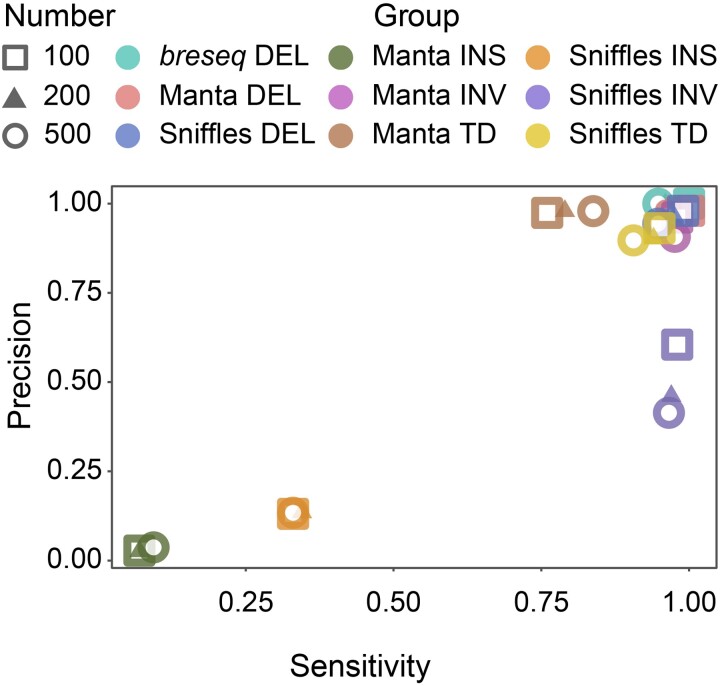
The sensitivity versus precision of SV detection for the 12 simulated data sets. Among them, *breseq* and Manta indicate results from short-read data sets, and Sniffles represents results from long-read data sets.

We first evaluated the reliability of the widely used SV detection pipelines by running them on simulated short-read and long-read data sets with mock mutation preset (see details in *Materials and Methods*). For the simulated short-read data set, *breseq* (v-0.35.1) performs the best for analyzing deletions, with sensitivity and precision both close to 100% (table 1, fig. 1, and supplementary tables S14 and S15, Supplementary Material online). Considering that *breseq* is mainly used to identify deletions and insertions mediated by mobile elements, we also used Manta (v-1.6.0) to detect other SVs besides deletions, such as insertions, tandem duplications, and inversions. The analysis achieved satisfying results for the precision of tandem duplications and sensitivity of inversions (tables 1 and 2, fig. 1, and supplementary tables S14 and S15, Supplementary Material online). Similarly, for the simulated long-read data set, Sniffles (v-1.0.12) was chosen because it outperformed other programs in SV detection, as shown in the testing results of different SV callers (supplementary tables S15 and S16, Supplementary Material online) (Sedlazeck et al. 2018; Liu et al. 2020; Okazaki et al. 2022), especially for deletions and tandem duplications (fig. 1, tables 1 and 2, and supplementary tables S14, and S15, Supplementary Material online). SV analyses on simulated data show that *breseq* detects deletions with high sensitivity and precision, Manta performs ideally on other SV types with short reads as input, and Sniffles is appropriate for detecting SVs using long-read sequencing (table 1, fig. 1, and supplementary tables S14 and S15, Supplementary Material online). The SV results from long-read sequencing are more reliable than those from short-read sequencing, as shown by the universally high F1 scores of most types of SVs (table 1 and supplementary table S17, Supplementary Material online), which is consistent with previous studies (Merker et al. 2018; Lesack et al. 2022). We also find that the number of SVs of certain types in the genome can affect the performance of the software to some extent. For example, using the short-read pipeline, sensitivity tends to increase with more tandem duplications, whereas for the long-read pipeline, the increase of inversion will greatly reduce the sensitivity and precision (fig. 1). Besides, we also note that even short-read sequencing can give highly reliable results for deletions and short inversions in simulated genomes. We then finalize the pipelines and use them on the Illumina and Nanopore sequences of the MA lines we ran.

**Table 1:** The Precision, Sensitivity, and F1 Score of Different SV Callers Using Simulated Data Sets. Mean and SE are the Mean and SE of the Above Measures for the 3 Simulated Genomes

		Insertion		Deletion		Tandem Duplication		Inversion	
Evaluator	Caller	Mean	SE	Mean	SE	Mean	SE	Mean	SE
**Precision**	*breseq*	0.3990	0.0513	1.0000	0	—	—	—	—
	Manta	0.0309	0.0021	0.9863	0.0031	0.9782	0.0015	0.9412	0.0121
	Sniffles	0.1337	0.0012	0.9633	0.0072	0.9107	0.0074	0.4941	0.0404
**Sensitivity**	*breseq*	0.0220	0.0042	0.9693	0.0111	—	—	—	—
	Manta	0.0780	0.0057	0.9757	0.0086	0.7960	0.0161	0.9770	0.0011
	Sniffles	0.3367	0.0031	0.9710	0.0087	0.9320	0.0094	0.9720	0.0029
**F1 score**	*breseq*	0.0417	0.0077	0.9843	0.0057	—	—	—	—
	Manta	0.0443	0.0031	0.9808	0.0036	0.8774	0.0100	0.9586	0.0066
	Sniffles	0.1920	0.0013	0.9671	0.0080	0.9212	0.0080	0.6516	0.0354

**Table 2:** The SV callers Used for Different Sequencing Platforms and SV Types

SV Types	Data Sets	Sequencing Platforms	SV Callers
Insertion	Simulation	Illumina	*breseq* + Manta
Deletion			*breseq* + Manta
Tandem duplication			Manta
Inversion			Manta
Insertion		Nanopore	Sniffles
Deletion			Sniffles
Tandem duplication			Sniffles
Inversion			Sniffles
Insertion	Real data	Illumina	*breseq* + Manta
Deletion			*breseq* + Manta
Tandem duplication			Manta
Inversion			Manta
Insertion		Nanopore	Sniffles
Deletion			Sniffles
Tandem duplication			Sniffles
Inversion			Sniffles

In addition, to ensure the transferability of the analysis pipeline for the simulated data, we similarly set up and analyzed the 0-variant mock genome. Based on the same short-read and long-read analysis pipelines, we did not detect any SVs, which confirmed the reliability of our pipelines.

### Genomic SV Rate of *E. coli* Based on Nanopore and/or Illumina Sequencing

**Fig. 2.**
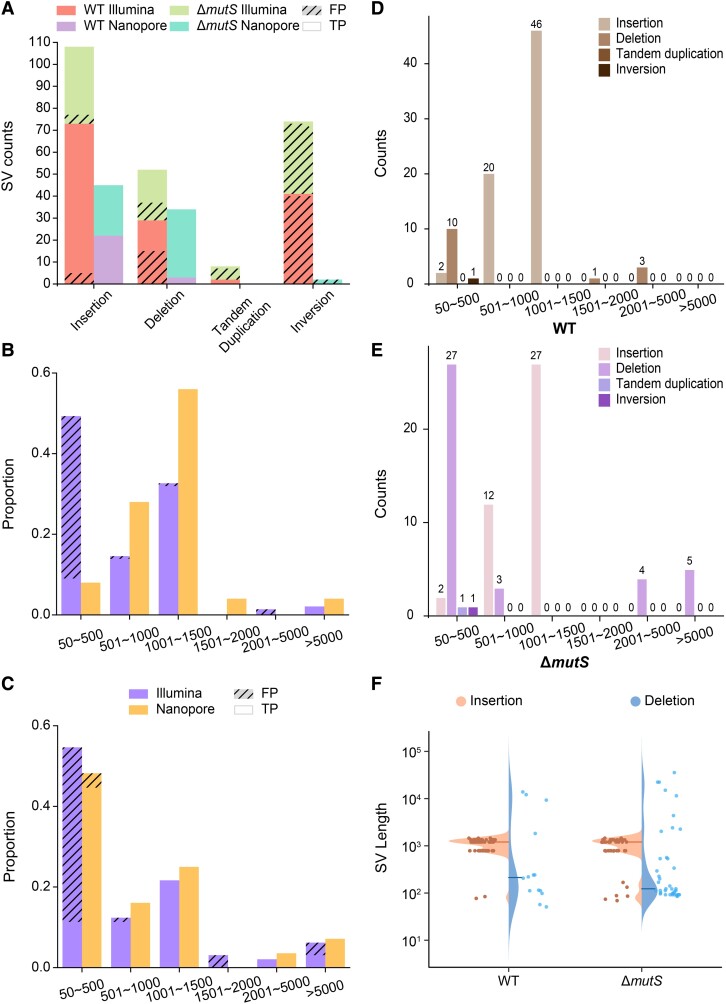
SVs detected in the WT and the Δ*mutS* MA lines. (*A*) True positives and false positives of the 4 types of SVs from different sequencing strategies. (*B*) and (*C*) True and false positives categorized by different lengths. (*D*) and (*E*) Length distribution of 4 types of SVs in the WT and Δ*mutS* lines. (*F*) Length-distribution of insertions and deletions SVs (The left-hand side of the violin plot, insertion; The right-hand side of the violin plot, deletion), and bold lines are the medians.

We applied *breseq* and Manta to detect SVs, using the Illumina PE150 sequences of the final-evolved 67 WT and 37 Δ*mutS* MA lines. Among these, 19 WT and 18 Δ*mutS* MA lines were also sequenced with a Nanopore PromethION sequencer, and SVs were detected with Sniffles (supplementary table S4, Supplementary Material online, and table 2). For the SVs detected by the short-read pipelines, 82 (56.9%) out of 144 for the WT and 48 (49.5%) out of 97 for the Δ*mutS* are verified; 25 (100%) out of 25 for the WT and 54 (96.4%) out of 56 for the Δ*mutS* with the pipelines for long-read sequencing are confirmed (tables 3 and 4 and supplementary table S18, Supplementary Material online). For short-read pipelines, the mean of true-positive SVs per WT or Δ*mutS* MA line is 1.22 or 1.23, respectively, and for long-read pipelines, 1.32 per WT line and 3.00 per Δ*mutS* line (table 3). Compared with the total number of SVs from the short-read pipelines, those detected by the Nanopore sequencing pipelines are small because only part of the MA lines were randomly chosen for costs concern. Consistent with the results from simulated data, the high validation rate and number of SVs from the Nanopore data demonstrate the superiority of long-read sequencing in SV detection. This is in strong contrast to the ultra-high false-positive rate of inversions and tandem duplications from short-read sequencing (fig. 2*A*). Nonetheless, the precision for Sniffles detecting insertions remains low (8.24% for WT and 6.00% for Δ*mutS*), even with the long-read strategy (supplementary table S17, Supplementary Material online). In addition, we also find that the medium- and long-length SVs, especially the insertions and deletions, are preferably detected, whereas the false-positive rate of the short SVs is relatively high (fig. 2*B* and 2C and supplementary tables S19 and S20, Supplementary Material online). Specifically, for SVs with different length ranges, the false-positive rates based on the short-read strategy are higher than those from the long-read strategy, especially for short and long SVs (fig. 2*B*and 2*C*). Finally, we combine SV results based on the 2 sequencing platforms and find that 83 out of 146 and 82 out of 133 SVs are validated in the WT and the Δ*mutS* MA lines, respectively (supplementary tables S19 and S20, Supplementary Material online). The number of SVs per WT or Δ*mutS* line, after combining SV results from the short-read and long-read strategies, is 1.24 or 2.22. The vast majority of these true-positive SVs are shorter than 1,500 bp in *E. coli* (fig. 2*D*and 2*E*).

**Table 3:** The SV Detection Results of the WT and the Δ*mutS* MA Lines Using Different Sequencing/Analytical Strategies

	WT		Δ*mutS*	
SV Categories	True Positive	False Positive	True Positive	False Positive
**Illumina**	**82**	**62**	**48**	**49**
Mean per line	1.22	0.92	1.23	1.32
Insertion	68	5	31	4
Deletion	13	15	15	8
Tandem duplication	0	2	1	5
Inversion	1	40	1	32
**Nanopore**	**25**	**0**	**54**	**2**
Mean per line	1.32	0	3.00	0.11
Insertion	22	0	23	0
Deletion	3	0	31	0
Tandem duplication	0	0	0	0
Inversion	0	0	0	2

Because Illumina-sequenced lines (67 WT, 37 Δ*mutS*; supplementary Tables S2 and S3, Supplementary Material online) were partially sequenced with the Nanopore platform (19 WT, 18 Δ*mutS*; supplementary Table S4, Supplementary Material online), the total number of True Positives or False Positives from Nanopore is lower than that from Illumina. The bold values represent the total number of four SVs types (Insertion, deletion, tandem duplication and inversion) with different sequencing strategy.

**Table 4:** The Details of Each Type of SVs in the WT and the Δ*mutS* MA Lines

SV Types or Status	WT	Δ*mutS*
**Insertion**	**68**	**41**
Mean per line	1.01	1.11
IS insertion	66	39
Non-IS insertion	2	2
**Deletion**	**14**	**39**
Mean per line	0.21	1.05
IS deletion	4	4
Non-IS deletion	10	35
**Tandem duplication**	**0**	**1**
Mean per line	0	0.03
**Inversion**	**1**	**1**
Mean per line	0.01	0.03
**IS-mediated SV rate**	**2.32 × 10^−4^**	**2.69 × 10^−4^**
95% CI for Poisson	1.82–2.95 × 10^−4^	1.95–3.62 × 10^−4^
**IS insertion rate**	**2.20 × 10^−4^**	**2.44 × 10^−4^**
95% CI for Poisson	1.70–2.80 × 10^−4^	1.74–3.34 × 10^−4^
**IS deletion rate**	**1.33 × 10^−5^**	**2.50 × 10^−5^**
95% CI for Poisson	0.36–3.41 × 10^−5^	0.68–6.41 × 10^−5^
**Total insertion length**	**74,356 bp**	**44,242 bp**
**Total deletion length**	**38,568 bp**	**122,942 bp**

The bold values represent the total number of four SVs types (Insertion, deletion, tandem duplication and inversion) with different sequencing strategy.

Based on the verified SVs, we calculate the genomic SV rate of the WT *E. coli* to be 2.77 × 10^−4^ per genome per cell division (95% CI: 2.95–4.34 × 10^−4^) and 5.26 × 10^−4^ per genome per cell division for the Δ*mutS* (95% CI: 7.37–10.34 × 10^−4^), with significant difference between the SV rates of the 2 strains—a sign of MMR influencing the major types of SVs (supplementary tables S21 and S22, Supplementary Material online). The WT SV rate is lower but still comparable with those large chromosomal rearrangements of *E. coli* reported in previous studies implying a low false-positive rate of the sequencing and analytical pipelines (also confirmed by the above analyses on the simulated data sets) (Raeside et al. 2014). We calculate the BPS rates of the WT and the Δ*mutS* to be 9.00 × 10^−4^ and 8.12 × 10^−2^ per genome per cell division, respectively. The SV rates are thus ∼31% and 0.65% of the BPSs rates for the 2 strains, respectively, consistent with previous findings that large-scale mutations are usually less abundant than the small mutations (Pang et al. 2010).

### Features of *de novo* SVs of *E. coli*

Interestingly, we find insertion bias among large-scale SVs in the WT MA lines (INS_WT_/DEL_WT_ = 4.86, INS_Δ*mutS*_/DEL_Δ*mutS*_ = 1.05) (table 4, fig. 2*F*, and supplementary tables S19 and S20, Supplementary Material online). Such insertion bias of SVs is different from the deletion bias of small indels previously reported (Kuo and Ochman 2009; Lee et al. 2012; Long et al. 2016; Danneels et al. 2018; Long et al. 2018a; Loewenthal et al. 2021). One previous study on SVs of the same *E. coli* WT strain found that IS-mediated insertions were more common than deletions (Lee et al. 2016). However, the bias is reversed by the SVs length in the Δ*mutS* MA lines, as the total length of deletions is about 2.78 times higher than that of the insertions (supplementary tables S19 and S20, Supplementary Material online). Consistent with small indels, this deletion bias in DNA length could be related to the genomic contraction in bacteria, especially for those hosted in other organisms (Gregory 2004; Merhej et al. 2009; Bobay and Ochman 2017). Besides, we also analyzed the distribution of SVs along the chromosome. For the WT, the distribution of insertions in the genome is approximate to uniform distribution, and the deletions mainly cluster in 0–0.8 Mbp and 2–4 Mbp regions (supplementary table S23, Supplementary Material online). And for Δ*mutS*, insertions have a trend to cluster in 0.2–0.6 Mbp and > 3.6 Mbp regions and deletions in 1.2–2.4 Mbp and >4.0 Mbp regions.

**Fig. 3.**
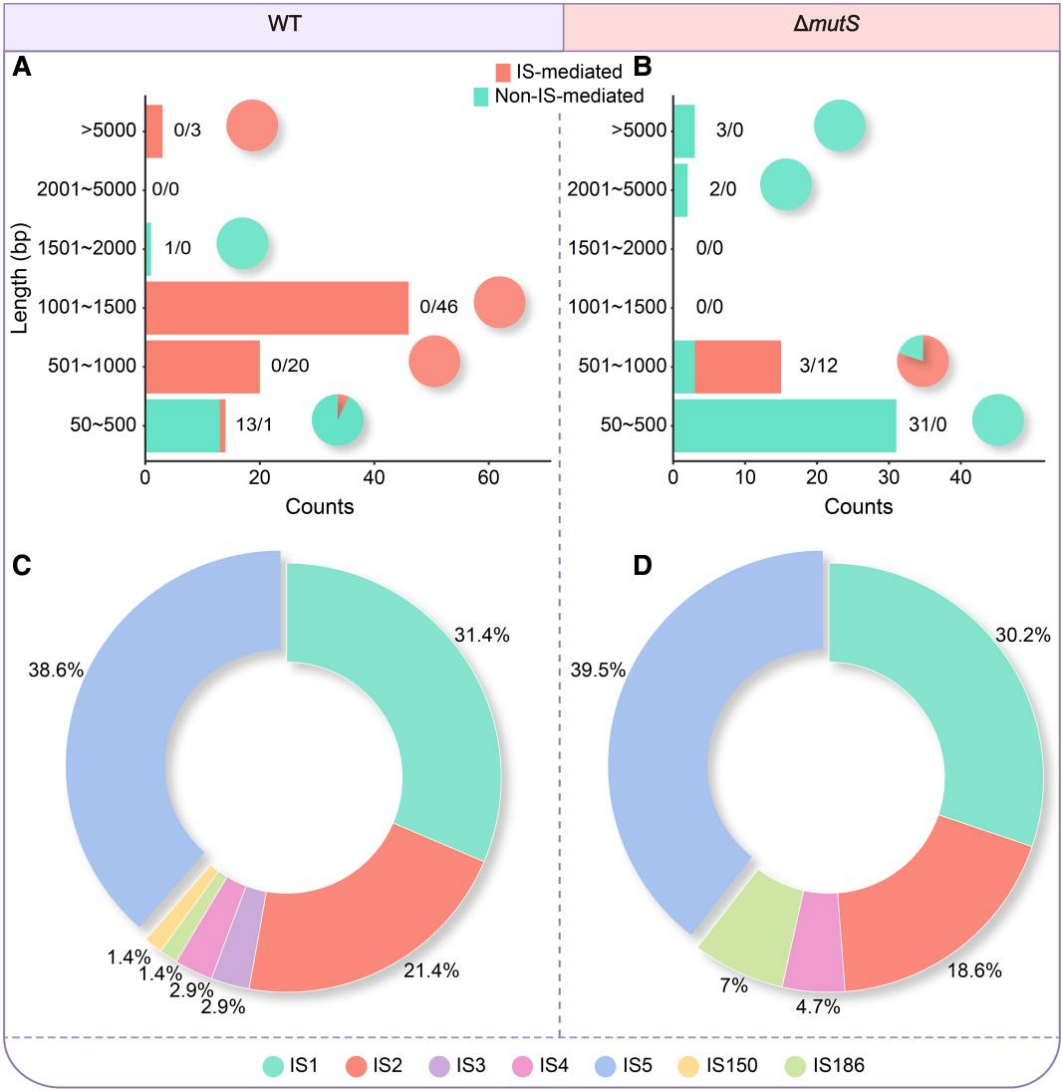
The IS element–associated SVs of the WT and the Δ*mutS* MA lines. (*A*) and (*B*) IS-mediated SVs versus non–IS-mediated ones. The pie chart in each length range shows the proportions of IS-mediated and non–IS-mediated SVs. The proportion of different IS element–associated SVs are shown in (*C*) and (*D*).

We also evaluated the features of IS element–mediated SVs—the most common SVs in bacterial genomes—in detail. IS elements are common mobile genetic elements in bacteria and play key roles in bacterial genome diversity and evolution (Ooka et al. 2009). Some SVs and complex recombination events mediated by IS elements have been found in *E. coli* MA lines (Lee et al. 2014; Raeside et al. 2014; Long et al. 2016). In our data sets, IS-mediated SVs dominate other SVs in both the WT and the Δ*mutS* MA lines, 70 (84.34%) and 43 (52.44%), respectively (table 4). The lengths of the IS-mediated SVs are extremely enriched around 500–1,000 bp (fig. 3*A*and 3*B* and supplementary tables S19 and S20, Supplementary Material online). There is no significant difference in the IS-mediated SV rate between the WT and the Δ*mutS* MA lines.

**Fig. 4.**
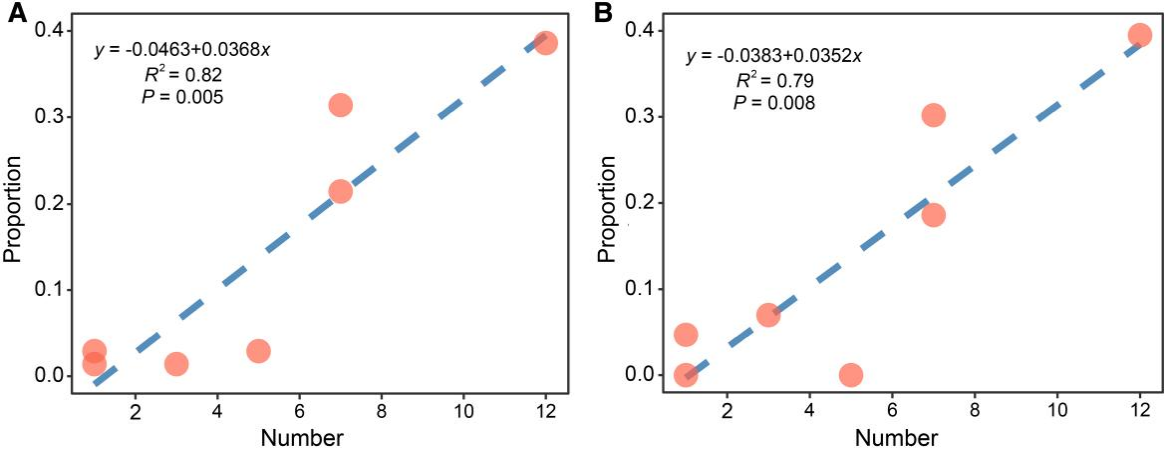
The correlation between the copy number of IS elements in the reference genome and their proportion out of all IS elements mediating SVs in the WT and the Δ*mutS* MA lines.

The IS element–mediated insertion rates of the WT (2.20 × 10^−4^ per genome per cell division) and the Δ*mutS E. coli* (2.44 × 10^−4^ per genome per cell division) (table 4) are comparable with those reported in previous studies, for example, 3.5 × 10^−4^ (95% CI: 3.2 × 10^−4^–3.7 × 10^−4^) per genome per cell division in the same *E. coli* strains (Sawyer et al. 1987; Lee et al. 2016; Vandecraen et al. 2017; Consuegra et al. 2021). Among the IS-mediated SVs in the WT and the Δ*mutS* MA lines, transpositions by IS5, IS1, and IS2 have the top 3 rankings, with IS5 elements accounting for ∼40% (fig. 3*C* and *D* and supplementary tables S19 and S20, Supplementary Material online). IS5 elements can insert the upstream or downstream of some operons to activate the expression of flagellar genes and glycoside metabolizing genes and thus indirectly alter the motility and glycoside utilization of *E. coli* (Schnetz and Rak 1992; Barker et al. 2004; Martinez-Vaz et al. 2005; Strauch and Beutin 2006; Wang and Wood 2011). Therefore, the high insertion rate of IS5 elements may be important in the migration and the niche evolution of bacteria. In addition, we find a significant correlation between the proportion of 1 type of IS elements (out of all IS elements mediating SVs) and their copy numbers in the reference genome (fig. 4). In other words, the more IS elements of the same type in the genome, the more frequently they will mediate SVs.

## Discussion

In this study, *de novo* spontaneous mutations of *E. coli* MG1655, especially the SVs, are extensively studied via different sequencing and analytical strategies. We analyze 104 final MA lines, including 67 WT and 37 Δ*mutS* lines. The mutation rates of BPSs and small indels are highly consistent with previous studies (supplementary tables S2, S3, and S13, Supplementary Material online) (Lee et al. 2012; Foster et al. 2015; Long et al. 2016). For the SV detection, we conclude that the strategy based on long-read sequencing and analysis is generally superior to that based on short reads in both simulated and real data (figs. 1 and 2*A–C*, tables 1 and 3, and supplementary tables S14, S15, and S18–S20, Supplementary Material online). The SV rates are 2.77 × 10^−4^ per genome per cell division in the WT and 5.26 × 10^−4^ in the Δ*mutS*, which are comparable with those previously reported (Lee et al. 2016). However, it is impossible to simulate all possible SV scenarios, and the complexity of real genomic regions can affect the precision for detecting SVs (Dierckxsens et al. 2021). Therefore, when applying the pipelines tested with simulated data to real data sets, the choices of software and parameters still need to be carefully refined.

Based on the simulated and the real data analyses, long-read sequencing is indeed more powerful in detecting all types of bacterial SVs, with high precision and accuracy compared with short-read sequencing (figs. 1 and 2*A–C*, tables 1 and 3, and supplementary tables S14, S15, and S18–S20, Supplementary Material online). Although the number of *de novo* SVs generated during the MA experiments is much smaller than those reported in studies on existing SVs in natural lineages, the high precision and accuracy of the long-read sequencing in SV detection are highly consistent (He et al. 2019; Mahmoud et al. 2019; Mantere et al. 2019; Chawla et al. 2021; Sakamoto et al. 2021). Analyses based on short reads show high SV false-positive rates in bacteria, because most software were initially developed for the human genome and their algorithms ignore some SVs in simple repetitive regions in order to save computation resources (Rausch et al. 2012; Fan et al. 2014; Layer et al. 2014; Deatherage et al. 2015; Chen et al. 2016). Nonetheless, *breseq* and Manta are still useful in detecting deletions and other SVs, although Manta works at the cost of a high rate of false positives (figs. 1 and 2*A–C* and supplementary tables S14, S19, and S20, Supplementary Material online). As previously reported, the limitation of short-read sequencing in SV detection could originate from the nearby BPSs or indels around the SV breakpoints (Cameron et al. 2019). Even integrating multiple callers, false positives are still common, and its high sensitivity comes at the cost of disproportionately lower precision, that is, sacrificing precision to improve sensitivity (Cameron et al. 2019; Mahmoud et al. 2019).

We applied 2 alternative strategies to detect SVs in simulated and real MA data: short-read–based sequencing and calling with *breseq* and Manta and long-read–based sequencing and calling with Sniffles. Apparently, different SV types are most amenable to different strategies regardless of the sequencing platforms. Compared with short-read–based methods, the long-read–based strategy performs better in the insertion SVs and will support future research related to SV characteristics and functions (table 1, fig. 2*A*, and supplementary tables S14 and S18–S20, Supplementary Material online). For identifying insertions, the advantages also apply to eukaryotes, suggesting that the ability of long reads to span longer repetitive regions and so outperforms short-read strategies (Cretu Stancu et al. 2017; Huddleston et al. 2017; Wong et al. 2018; Liu et al. 2020; Zhao et al. 2021b). For the deletion, although short-read sequencing performed well for SV detection in simulation results, long-read sequencing has higher accuracy with real data (table 1, fig. 2*A*, and supplementary tables S14 and S18–S20, Supplementary Material online). This may be due to the high complexity of the real situation and may benefit from the advantage of long-read sequencing even with low coverage in previous research (Kosugi et al. 2019). Tandem duplications and inversions are rare in the real data sets (fig. 2*A* and supplementary tables S14 and S18–S20, Supplementary Material online), suggesting that there are relatively few tandem duplication and inversion SVs in the MA lines. These results corroborate that the 2 strategies can be combined for thorough SV detection and even short-read sequencing could be accurate enough using *breseq* and Manta, if deletion SV is considered only.

Previous studies on bacterial MA have primarily focused on characterizing BPSs and indels, and only limited inference about SVs based on short-read sequencing is available (Foster et al. 2015; Long et al. 2015; Kucukyildirim et al. 2016; Long et al. 2016; Strauss et al. 2017; Tincher et al. 2017; Long et al. 2018a; Pan et al. 2021; Wu et al. 2021). The SV detection strategy based on long reads has been generating numerous reliable results, for example, in the metagenomic study of lake bacterioplanktons and for detecting potential large-scale assembly errors of complex bacterial genomes with long repeat regions (Schmid et al. 2018; Okazaki et al. 2022). Our results also indicate that long-read sequencing, long-read tools, and intensive SV candidate validation with Sanger sequencing are needed to fully characterize full-scale mutations in evolved MA lines (fig. 2*A*–*C* and supplementary tables S18–S20, Supplementary Material online).

However, although long-read detection tools have advantages over short-read ones when applied to both simulated and real bacterial data for identifying SVs, there are still some issues. Because long-read sequencing has a high error rate, it can affect the efficiency of long-read tools to detect SVs (Jiang et al. 2021). In addition, SV detection using long-read tools is also affected by the sequencing depth and SV types, for example, high sequencing depth could even reduce the accuracy of some tools (Luan et al. 2020; Dierckxsens et al. 2021; Lesack et al. 2022). Similarly, long-read tools also detect inversions unsatisfactorily, which also needs facilitation of other algorithms (Parrish et al. 2013).

The strategies outlined in this study should facilitate future research that involves SV analyses. For example, studies on gut microbiomes have shown that unique SVs can represent the genetic fingerprints of specific communities (Chen et al. 2021). The total length of SVs is almost 10× that of BPSs in our study, also demonstrating the important role of SVs in genome evolution (Korbel et al. 2007; Escaramís et al. 2015; Hämälä et al. 2021). In addition, SVs are reported to be closely associated with bacterial growth and adaptation to the environment, and their changes can also alter the immunity and metabolism of the host (Zeevi et al. 2019; Wang et al. 2021). It has also been shown that IS-mediated SVs in a population can not only promote evolution but also limit evolution after a meltdown (Consuegra et al. 2021). Advanced sequencing technologies combined with sophisticated programs would eventually push the precision and accuracy of SV detection to the point that would satisfy most biological studies. Further studies are needed in the future regarding the distribution of SV fitness effects in bacteria, and such studies would provide more insights into long-term genome evolution.

## Materials and Methods

### Strains and MA Procedures

All *Escherichia coli* strains were in the K-12 MG1655 background and generously provided by Patricia Foster's lab. Eighty WT and 40 Δ*mutS* MA lines were initiated and cultured on LB agar (Solarbio, Cat. No.: L8290) at 37 °C. Each line was single-colony transferred daily. We transferred each MA line 160 times on average, taking more than 5 months. In order to estimate the cell divisions between transfers (*Num*) by the colony-forming-units, we performed serial dilution every 10 days, by randomly choosing and razor-cutting a single colony from each of the 5 lines for the WT and the Δ*mutS* MA lines, respectively. Based on the formula log_2_(*Num*), there were, on average, 28 cell divisions for the WT lines and 27 for the Δ*mutS* lines between 2 adjacent transfers.

### DNA Extraction, Library Construction, and Genome Sequencing

After the last transfer, we picked a single colony for each final MA line as well as the ancestral line for each strain and cultured them in the LB broth (Solarbio, Cat. No.: L8291) in quadruplicate overnight at 37 °C. One of the 4 cultures was used to extract DNA with MasterPure^TM^ Complete DNA and RNA Purification Kit (Lucigen, Cat No.: MC85200) for Illumina sequencing. Each of the remaining 3 replicates was mixed with glycerin (10%) and stored at −80 °C. We constructed the short-read libraries of DNA that passed the concentration and quality requirements using an optimized protocol for TruePrep® DNA Library Prep Kit V2 for Illumina (Vazyme, Cat. No.: TD501-01) and TruePrep® Index Kit V3 for Illumina (Vazyme, Cat. No.: TD203). After agarose gel electrophoresis and cutting the target bands to recycle with the E.Z.N.A.® Gel Extraction Kit (Omega Bio-tek, Cat. No.: D2500-02), we obtained the libraries with insert sizes of about 300 bp. Then, PE150 sequencing was performed using 1 Illumina NovaSeq6000 sequencer at Berry Genomics, Beijing. For the WT and the Δ*mutS* final MA lines, we randomly chose 19 lines from each group, as well as their ancestors, to extract DNA and construct the libraries for the Nanopore long-read sequencing. The standardized mixed libraries were pooled and loaded into 1 flow cell (R9.4) and sequenced with 1 Oxford Nanopore PromethION sequencer (Benagen, Wuhan, China). Then, the electrical signals were converted into DNA bases by Guppy (v-5.0.16). Next, the adapters were removed from the data and the data was filtered with Q ≥ 7. After quality control, about 1–3 Gbp sequences for each sample were finally obtained (supplementary table S4, Supplementary Material online).

### BPS and Indel Mutation Analysis

For the Illumina sequencing data, the 2 × 150 bp paired-end reads were first trimmed by Fastp (v-0.20) (Chen et al. 2018) to remove adapters and low-quality reads. After trimming, the reads were mapped to the reference genome (NC_000913.3), using the “mem” function in Burrows–Wheeler Aligner (v-0.7.17) (Li and Durbin 2009). The mapped reads were in SAM format and transformed into BAM format by SAMtools (v-1.9) (Li et al. 2009). Duplicate reads were removed by the function MarkDuplicates of picard-tools (v-2.20.1). Based on the local re-assembly feature, we used the HaplotypeCaller of Genome Analysis Toolkit (GATK, v-4.1.2.0) (McKenna et al. 2010; DePristo et al. 2011; Van der Auwera et al. 2013) with standard hard filters to call the BPSs and indels. Therefore, 13 lines were removed because of low coverage (less than 20×), cross-contamination of sequenced lines (randomly removing 1 line if 2 lines shared exactly the same BPS in the same site), or carrying mutations on repair genes (supplementary table S1, Supplementary Material online), and eventually 67 WT and 37 Δ*mutS* MA lines were used in the final analyses. All the indels were manually curated with the Integrative Genomics Viewer (IGV, v-2.8.2) (Thorvaldsdóttir et al. 2012).

Using the filtered BPSs and indels, we calculated the mutation rate *μ* with the formula:


$$\mu =\frac{m}{\sum_{1}^{n}\;N\times T}$$

Here, *n* was the number of MA lines. The number of mutations for all MA lines, the analyzed sites for each MA line, and the total cell divisions during the transfers were denoted by *m*, *N*, and *T*, respectively. The context-dependent mutation rates were analyzed as in our previous study (Long et al. 2015).

### *E. coli* Genome Simulation

In order to evaluate the SV detection pipelines and based on the reference genome of *E. coli* MG1655 (NC_000913.3), we established 4 groups of simulated genomes, each carrying known SVs of only 1 type: insertions, deletions, tandem duplications, or inversions. Each group contained 3 simulated genomes with 100, 200, or 500 known SVs. This was done using RSVSim (v-1.34.0) (Bartenhagen and Dugas 2013), a Bioconductor package in R. In addition, we also randomly simulated BPSs and indels near the breakpoints of these SVs, mainly distributed in the range of 100 bp upstream or downstream of the breakpoints. The percentages of BPSs and indels out of the total number of SVs within a breakpoint's flanking regions are 0.1% and 0.05%, respectively, and the maximum length of indels is 20 bp. According to RSVSim's built-in algorithms, 1 flanking region can contain at most 1 indel and breakpoints’coordinates of SVs in the genome follow a uniform distribution.

The SV lengths in the simulated genomes of the 4 groups were set from 50 to 10,000 bp, with SV length distribution of 70% 50–1,000 bp, 20% 1,001–5,000 bp, and 10% 5,001–100,000 bp. Within the range, the length of each specific SV is randomly generated in R (v-4.1.2) (R Core Team 2016). The details and statistics of the introduced SVs of the simulated genomes are in supplementary figure S3 and supplementary tables S24 and S25, Supplementary Material online. Supplementary files S2–S13, Supplementary Material online, are simulated genomes, with detailed information in supplementary table S26, Supplementary Material online.

Besides, we also simulated a 0-variant genome and its short- and long-read sequencing data, and the methods as well as parameters were consistent with those described above.

### Simulation of Illumina Short Reads and Nanopore Long Reads

ART (v-2.5.8) (Huang et al. 2011) simulated the short-read data sets using the above simulated genomes with known SVs. These data sets were composed of 2 × 150 bp Illumina short reads with a mean sequencing depth of about 100×, and the mean and standard deviation for the insert sizes were 300 and 50 bp.

The long-read data sets were simulated by Badread (v-0.2.0) (Wick 2019) with the following recommended parameters for the best simulation data set: –quantity 200 × –error_model nanopore2020 –qscore_model ideal –glitches 0,0,0 –random_read 0 –chimeras 0 –junk_reads 0 –identity 95,100,4 –start_adapter_seq“” –end_adapter_seq “”. With these, we acquired the FASTQ files with high-quality scores.

The simulated short-read and long-read data sets were uploaded to the NCBI SRA database (BioProject Number: PRJNA856428).

### Testing the Pipelines by Detecting SVs in the Simulated Data Sets

Using the simulated data sets, we applied different analytical pipelines to identify SVs. We first performed quality controls on the simulated data sets. For the Illumina data sets, the process for obtaining the BAM files is the same as the above *BPS and Indel Mutation Analysis* section. For the Nanopore data sets, they were firstly filtered by NanoFilt (v-2.8.0) (De Coster et al. 2018) to keep the reads with quality score Q ≥ 7 and then corrected by canu (v-1.7.1) (Koren et al. 2017). Then, the corrected reads were mapped to the reference genome NC_000913.3 using NGMLR(v-0.2.7) (Sedlazeck et al. 2018). Next, the SAM format files were converted into BAM files and sorted using SAMtools.

The pipelines with *breseq* (v-0.35.1) (Barrick et al. 2014; Deatherage and Barrick 2014) and Manta (v-1.6.0) (Chen et al. 2016) were used to identify SVs using the preprocessed short-read data sets. *breseq* (Barrick et al. 2014; Deatherage and Barrick 2014) was a versatile tool that could mainly detect IS-mediated insertions and deletions of haploid microbial genomes. Given that *breseq* could not detect non–IS-mediated insertions and the random simulation introduces IS-mediated insertions at low frequency (Barrick et al. 2014), we only used *breseq* for insertion and deletion calling. *breseq* was used to map the clean short reads to the reference genome by BOWTIE2, then implement the split-read alignment methods, reconstruct the candidate junction sequences into a new reference, and map again to predict and annotate mutations after correcting and analyzing with the default parameters. As *breseq* is mainly used for detecting deletions and insertions mediated by mobile elements, we also used Manta to complement the limitations in detecting other types of SVs (insertions, tandem duplications, and inversions). Manta performs excellently in detecting SVs in human genomes based on short reads (Cameron et al. 2019).

The other pipeline was based on Sniffles (v-1.0.12) (Sedlazeck et al. 2018). We required the number of supporting reads ≥ 10 and the SV length ≥ 50 bp, with default values for other parameters. In addition, we also used NanoVar (v-1.3.8) (Tham et al. 2020) and NanoSV (v-1.2.4) (Cretu Stancu et al. 2017) to detect the SVs in the simulated data sets and then compared these results with Sniffles to choose the best-performance pipeline.

To evaluate the detection efficiency of each pipeline, we introduced 3 criteria: sensitivity, precision, and F1 score. The calculations of these values follow the confusion matrix rule. After calculating the true positives (TP), false negatives (FN), and false positives (FP), we used the formula as follows:


$$\mathrm{sensitivity}=\frac{\mathrm{TP}}{\mathrm{TP}+\mathrm{FN}}$$

$$\mathrm{precision}=\frac{\mathrm{TP}}{\mathrm{TP}+\mathrm{FP}}$$

$$F1\;\mathrm{score}=2\;*\;\frac{\mathrm{sensitivity}\;*\;\mathrm{precision}}{\mathrm{sensitivity}+\mathrm{precision}}$$

The True Positives needed to meet 3 conditions: 1) the type of SVs must be the same as the simulated one, 2) the start position for called SV is the same as or within ±30 bp of the corresponding simulated SV, and 3) the SV length differs from the simulated one by no more than 30%. The error distribution associated with these cutoff lines is also shown in supplementary table S27, Supplementary Material online. Failure to meet any condition would be considered as 1 false-positive SV.

### The Detection of SVs in the Real Data from MA Lines

To identify SVs in the short-read sequenced MA lines (supplementary tables S2 and S3, Supplementary Material online), we used *breseq* to detect IS-mediated insertions and deletions and retained all types of SVs called by Manta as a complement for the *breseq* results. The SVs, called by the 2 software, were combined as the candidate calls. For the Nanopore-sequenced MA lines (supplementary table S4, Supplementary Material online), the same Sniffles parameters as those used on the simulated data sets were performed. We subsequently eliminated the SVs existed in the ancestors from the candidate SV calls. Then, SVs detected in 3 or more MA lines in each set (either long- or short-read) were also removed.

### PCR Validation of Candidate SVs

Before Sanger sequencing, we filtered out hundreds of false positives in MA lines that were also present in the ancestral line (SVs were called by Sniffles if there are sequence difference between the ancestral genome and the reference genome) and those labeled as “imprecise” by Sniffles (supplementary table S17, Supplementary Material online, FP in MA-WT and MA-Δ*mutS*). The SV calls after the above filtering were then validated by PCR, using Primer5.0 to design primers for each specific target region and BlastN (v-2.13.0) (Zhang et al. 2000) to confirm that primers were unique with low similarity to other nontarget genomic regions. All the primer sequences are shown in supplementary table S28, Supplementary Material online. The designed primers were then used for PCR amplification and Sanger sequencing (Tsingke Biotechnology Co., Ltd., Qingdao, China). One SV was considered to be true positive if the Sanger sequences and the candidate call show the consistent SV type, start point difference <100 bp, and length difference <30%, which is set based on the error distribution (supplementary table S27, Supplementary Material online).

### Statistics and Plotting

Statistical tests were done in R (v-4.1.2) and JMP Pro (v-16.0.0), and plottings were done in ggplot2 (Wickham 2019) and OriginPro (v-2022).

## Supplementary Material

evad106_Supplementary_Data

## Data Availability

All available MA raw data in this study, namely Illumina and Nanopore FASTQ files, were uploaded to the NCBI SRA database (BioProject Number: PRJNA856428).

## References

[evad106-B1] Barker CS , Prüß BM, Matsumura P. 2004. Increased motility of *Escherichia coli* by insertion sequence element integration into the regulatory region of the flhD operon. J Bacteriol. 186(22):7529–7537. 15516564 10.1128/JB.186.22.7529-7537.2004 PMC524886

[evad106-B2] Barrick JE , et al 2014. Identifying structural variation in haploid microbial genomes from short-read resequencing data using breseq. BMC Genomics. 15(1):1039. 25432719 10.1186/1471-2164-15-1039 PMC4300727

[evad106-B3] Bartenhagen C , Dugas M. 2013. RSVSim: an R/Bioconductor package for the simulation of structural variations. Bioinformatics 29(13):1679–1681. 23620362 10.1093/bioinformatics/btt198

[evad106-B4] Bobay L-M , Ochman H. 2017. The evolution of bacterial genome architecture. Front Genet. 8:72. 28611826 10.3389/fgene.2017.00072 PMC5447742

[evad106-B5] Cameron DL , Di Stefano L, Papenfuss AT. 2019. Comprehensive evaluation and characterisation of short read general-purpose structural variant calling software. Nat Commun. 10(1):3240. 31324872 10.1038/s41467-019-11146-4 PMC6642177

[evad106-B6] Chan YF , et al 2010. Adaptive evolution of pelvic reduction in sticklebacks by recurrent deletion of a Pitx1 enhancer. Science 327(5963):302–305. 20007865 10.1126/science.1182213 PMC3109066

[evad106-B7] Chawla HS , et al 2021. Long-read sequencing reveals widespread intragenic structural variants in a recent allopolyploid crop plant. Plant Biotechnol J. 19(2):240–250. 32737959 10.1111/pbi.13456 PMC7868984

[evad106-B8] Chen X , et al 2016. Manta: rapid detection of structural variants and indels for germline and cancer sequencing applications. Bioinformatics 32(8):1220–1222. 26647377 10.1093/bioinformatics/btv710

[evad106-B9] Chen L , et al 2021. The long-term genetic stability and individual specificity of the human gut microbiome. Cell 184(9):2302–2315. e12. 33838112 10.1016/j.cell.2021.03.024

[evad106-B10] Chen L , et al 2022. Short- and long-read metagenomics expand individualized structural variations in gut microbiomes. Nat Commun. 13(1):3175. 35676264 10.1038/s41467-022-30857-9 PMC9177567

[evad106-B11] Chen S , Zhou Y, Chen Y, Gu J. 2018. . Fastp: an ultra-fast all-in-one FASTQ preprocessor. Bioinformatics 34(17):i884–i890. 30423086 10.1093/bioinformatics/bty560 PMC6129281

[evad106-B12] Consuegra J , et al 2021. Insertion-sequence-mediated mutations both promote and constrain evolvability during a long-term experiment with bacteria. Nat Commun. 12(1):980. 33579917 10.1038/s41467-021-21210-7 PMC7881107

[evad106-B13] Cretu Stancu M , et al 2017. Mapping and phasing of structural variation in patient genomes using nanopore sequencing. Nat Commun. 8(1):1326. 29109544 10.1038/s41467-017-01343-4 PMC5673902

[evad106-B14] Damkiær S , Yang L, Molin S, Jelsbak L. 2013. Evolutionary remodeling of global regulatory networks during long-term bacterial adaptation to human hosts. Proc Natl Acad Sci U S A. 110(19):7766–7771. 23610385 10.1073/pnas.1221466110 PMC3651418

[evad106-B15] Danneels B , Pinto-Carbó M, Carlier A. 2018. Patterns of nucleotide deletion and insertion inferred from bacterial pseudogenes. Genome Biol Evol. 10(7):1792–1802. 29982456 10.1093/gbe/evy140 PMC6054270

[evad106-B16] Deatherage DE , Barrick JE. 2014. Identification of mutations in laboratory-evolved microbes from next-generation sequencing data using breseq. In: Sun L and Shou W, editors. Engineering and analyzing multicellular systems. New York: Springer. p. 165–188. 10.1007/978-1-4939-0554-6_12 PMC4239701 24838886

[evad106-B17] Deatherage DE , Traverse CC, Wolf LN, Barrick JE. 2015. Detecting rare structural variation in evolving microbial populations from new sequence junctions using breseq. Front Genet. 5:468. 25653667 10.3389/fgene.2014.00468 PMC4301190

[evad106-B18] De Coster W , D’hert S, Schultz DT, Cruts M, Van Broeckhoven C. 2018. Nanopack: visualizing and processing long-read sequencing data. Bioinformatics 34(15):2666–2669. 29547981 10.1093/bioinformatics/bty149 PMC6061794

[evad106-B19] DePristo MA , et al 2011. A framework for variation discovery and genotyping using next-generation DNA sequencing data. Nat Genet. 43(5):491–498. 21478889 10.1038/ng.806 PMC3083463

[evad106-B20] Dierckxsens N , Li T, Vermeesch JR, Xie Z. 2021. A benchmark of structural variation detection by long reads through a realistic simulated model. Genome Biol. 22(1):342. 34911553 10.1186/s13059-021-02551-4 PMC8672642

[evad106-B21] Emerson J , Cardoso-Moreira M, Borevitz JO, Long M. 2008. Natural selection shapes genome-wide patterns of copy-number polymorphism in *Drosophila melanogaster*. Science 320(5883):1629–1631. 18535209 10.1126/science.1158078

[evad106-B22] Escaramís G , Docampo E, Rabionet R. 2015. A decade of structural variants: description, history and methods to detect structural variation. Briefings Funct Genomics. 14(5):305–314. 10.1093/bfgp/elv014 25877305

[evad106-B23] Fan X , Abbott TE, Larson D, Chen K. 2014. Breakdancer: identification of genomic structural variation from paired-end read mapping. Curr Protoc Bioinf. 45(1):15.6. 1–15.6. 11. 10.1002/0471250953.bi1506s45 PMC4138716 25152801

[evad106-B24] Foster PL . 2006. Methods for determining spontaneous mutation rates. Methods Enzymol. 409:195–213. 16793403 10.1016/S0076-6879(05)09012-9 PMC2041832

[evad106-B25] Foster PL , Lee H, Popodi E, Townes JP, Tang H. 2015. Determinants of spontaneous mutation in the bacterium *Escherichia coli* as revealed by whole-genome sequencing. Proc Natl Acad Sci U S A. 112(44):E5990–E5999. 26460006 10.1073/pnas.1512136112 PMC4640725

[evad106-B26] Gregory TR . 2004. Insertion–deletion biases and the evolution of genome size. Gene 324:15–34. 14693368 10.1016/j.gene.2003.09.030

[evad106-B27] Hämälä T , et al 2021. Genomic structural variants constrain and facilitate adaptation in natural populations of *Theobroma cacao*, the chocolate tree. Proc Natl Acad Sci U S A. 118(35):e2102914118. 10.1073/pnas.2102914118 PMC8536383 34408075

[evad106-B28] He Y , et al 2019. Long-read assembly of the Chinese rhesus macaque genome and identification of ape-specific structural variants. Nat Commun. 10(1):4233. 31530812 10.1038/s41467-019-12174-w PMC6749001

[evad106-B29] Huang W , Li L, Myers JR, Marth GT. 2011. ART: a next-generation sequencing read simulator. Bioinformatics 28(4):593–594. 22199392 10.1093/bioinformatics/btr708 PMC3278762

[evad106-B30] Huddleston J , et al 2017. Discovery and genotyping of structural variation from long-read haploid genome sequence data. Genome Res. 27(5):677–685. 27895111 10.1101/gr.214007.116 PMC5411763

[evad106-B31] Iqbal Z , Caccamo M, Turner I, Flicek P, McVean G. 2012. De novo assembly and genotyping of variants using colored De Bruijn graphs. Nat Genet. 44(2):226–232. 22231483 10.1038/ng.1028 PMC3272472

[evad106-B32] Iskow RC , Gokcumen O, Lee C. 2012. Exploring the role of copy number variants in human adaptation. Trends Genet. 28(6):245–257. 22483647 10.1016/j.tig.2012.03.002 PMC3533238

[evad106-B33] Iyer RR , Pluciennik A, Burdett V, Modrich PL. 2006. DNA mismatch repair: functions and mechanisms. Chem Rev. 106(2):302–323. 16464007 10.1021/cr0404794

[evad106-B34] Jiang T , et al 2021. Long-read sequencing settings for efficient structural variation detection based on comprehensive evaluation. BMC Bioinf. 22(1):552. 10.1186/s12859-021-04422-y PMC8588741 34772337

[evad106-B35] Kondrashov FA . 2012. Gene duplication as a mechanism of genomic adaptation to a changing environment. Proc Royal Soc B. 279(1749):5048–5057. 10.1098/rspb.2012.1108 PMC3497230 22977152

[evad106-B36] Konrad M , et al 1996. Large homozygous deletions of the 2q13 region are a major cause of juvenile nephronophthisis. Hum Mol Genet. 5(3):367–371. 8852662 10.1093/hmg/5.3.367

[evad106-B37] Korbel JO , et al 2007. Paired-end mapping reveals extensive structural variation in the human genome. Science 318(5849):420–426. 17901297 10.1126/science.1149504 PMC2674581

[evad106-B38] Koren S , et al 2017. Canu: scalable and accurate long-read assembly via adaptive k-mer weighting and repeat separation. Genome Res. 27(5):722–736. 28298431 10.1101/gr.215087.116 PMC5411767

[evad106-B39] Kosugi S , et al 2019. Comprehensive evaluation of structural variation detection algorithms for whole genome sequencing. Genome Biol. 20(1):117. 31159850 10.1186/s13059-019-1720-5 PMC6547561

[evad106-B40] Kucukyildirim S , et al 2016. The rate and spectrum of spontaneous mutations in *Mycobacterium smegmatis*, a bacterium naturally devoid of the postreplicative mismatch repair pathway. G3 6(7):2157–2163. 27194804 10.1534/g3.116.030130 PMC4938668

[evad106-B41] Kuo C-H , Ochman H. 2009. Deletional bias across the three domains of life. Genome Biol Evol. 1:145–152. 20333185 10.1093/gbe/evp016 PMC2817411

[evad106-B42] Layer RM , Chiang C, Quinlan AR, Hall IM. 2014. LUMPY: a probabilistic framework for structural variant discovery. Genome Biol. 15(6):R84.. 24970577 10.1186/gb-2014-15-6-r84 PMC4197822

[evad106-B43] Lee H , Doak TG, Popodi E, Foster PL, Tang H. 2016. Insertion sequence-caused large-scale rearrangements in the genome of *Escherichia coli*. Nucleic Acids Res. 44(15):7109–7119. 27431326 10.1093/nar/gkw647 PMC5009759

[evad106-B44] Lee H , Popodi E, Foster PL, Tang H. 2014. Detection of structural variants involving repetitive regions in the reference genome. J Comput Biol. 21(3):219–233. 24552580 10.1089/cmb.2013.0129

[evad106-B45] Lee H , Popodi E, Tang H, Foster PL. 2012. Rate and molecular spectrum of spontaneous mutations in the bacterium *Escherichia coli* as determined by whole-genome sequencing. Proc Natl Acad Sci U S A. 109(41):E2774–E2783. 22991466 10.1073/pnas.1210309109 PMC3478608

[evad106-B46] Lesack K , Mariene GM, Andersen EC, Wasmuth JD. 2022. Different structural variant prediction tools yield considerably different results in *Caenorhabditis elegans*. PLoS One. 17(12):e0278424. 36584177 10.1371/journal.pone.0278424 PMC9803319

[evad106-B47] Li H , et al 2009. The sequence alignment/map format and SAMtools. Bioinformatics 25(16):2078–2079. 19505943 10.1093/bioinformatics/btp352 PMC2723002

[evad106-B48] Li H , Durbin R. 2009. Fast and accurate short read alignment with Burrows-Wheeler transform. Bioinformatics 25(14):1754–1760. 19451168 10.1093/bioinformatics/btp324 PMC2705234

[evad106-B49] Lieberman TD , et al 2011. Parallel bacterial evolution within multiple patients identifies candidate pathogenicity genes. Nat Genet. 43(12):1275–1280. 22081229 10.1038/ng.997 PMC3245322

[evad106-B50] Liu Y , et al 2020. Comparison of multiple algorithms to reliably detect structural variants in pears. BMC Genomics. 21(1):61. 31959124 10.1186/s12864-020-6455-x PMC6972009

[evad106-B51] Loewenthal G , et al 2021. A probabilistic model for indel evolution: differentiating insertions from deletions. Mol Biol Evol. 38(12):5769–5781. 34469521 10.1093/molbev/msab266 PMC8662616

[evad106-B52] Long H , et al 2015. Mutation rate, spectrum, topology, and context-dependency in the DNA mismatch repair-deficient *Pseudomonas fluorescens* ATCC948. Genome Biol Evol. 7(1):262–271. 10.1093/gbe/evu284 PMC4316635 25539726

[evad106-B53] Long H , et al 2016. Antibiotic treatment enhances the genome-wide mutation rate of target cells. Proc Natl Acad Sci U S A. 113(18):E2498–E2505. 27091991 10.1073/pnas.1601208113 PMC4983809

[evad106-B54] Long H , et al 2018b. Evolutionary determinants of genome-wide nucleotide composition. Nat Ecol Evol. 2(2):237–240. 29292397 10.1038/s41559-017-0425-y PMC6855595

[evad106-B55] Long H , Miller SF, Williams E, Lynch M. 2018a. Specificity of the DNA mismatch repair system (MMR) and mutagenesis bias in bacteria. Mol Biol Evol. 35(10):2414–2421. 29939310 10.1093/molbev/msy134 PMC6188547

[evad106-B56] Luan M-W , Zhang X-M, Zhu Z-B, Chen Y, Xie S-Q. 2020. Evaluating structural variation detection tools for long-read sequencing datasets in Saccharomyces cerevisiae. Front Genet. 11:159. 32211024 10.3389/fgene.2020.00159 PMC7075250

[evad106-B57] Lupski JR , et al 1991. DNA duplication associated with Charcot-Marie-Tooth disease type 1A. Cell 66(2):219–232. 1677316 10.1016/0092-8674(91)90613-4

[evad106-B58] Lynch M , et al 2016. Genetic drift, selection and the evolution of the mutation rate. Nat Rev Genet. 17(11):704–714. 27739533 10.1038/nrg.2016.104

[evad106-B59] Ma Z , et al 2021. High-quality genome assembly and resequencing of modern cotton cultivars provide resources for crop improvement. Nat Genet. 53(9):1385–1391. 34373642 10.1038/s41588-021-00910-2 PMC8423627

[evad106-B60] Mahmoud M , et al 2019. Structural variant calling: the long and the short of it. Genome Biol. 20(1):246. 31747936 10.1186/s13059-019-1828-7 PMC6868818

[evad106-B61] Mantere T , Kersten S, Hoischen A. 2019. Long-read sequencing emerging in medical genetics. Front Genet. 10:426. 31134132 10.3389/fgene.2019.00426 PMC6514244

[evad106-B62] Martinez-Vaz BM , Xie Y, Pan W, Khodursky AB. 2005. Genome-wide localization of mobile elements: experimental, statistical and biological considerations. BMC Genomics. 6(1):81. 15929794 10.1186/1471-2164-6-81 PMC1174868

[evad106-B63] McKenna A , et al 2010. The Genome Analysis Toolkit: a MapReduce framework for analyzing next-generation DNA sequencing data. Genome Res. 20(9):1297–1303. 20644199 10.1101/gr.107524.110 PMC2928508

[evad106-B64] Merhej V , Royer-Carenzi M, Pontarotti P, Raoult D. 2009. Massive comparative genomic analysis reveals convergent evolution of specialized bacteria. Biol Direct. 4(1):13. 19361336 10.1186/1745-6150-4-13 PMC2688493

[evad106-B65] Merker JD , et al 2018. Long-read genome sequencing identifies causal structural variation in a Mendelian disease. Genet Med. 20(1):159–163. 28640241 10.1038/gim.2017.86 PMC5741540

[evad106-B66] Okazaki Y , Nakano SI, Toyoda A, Tamaki H. 2022. Long-read-resolved, ecosystem-wide exploration of nucleotide and structural microdiversity of lake bacterioplankton genomes. mSystems 7(4):e00433-22. 35938717 10.1128/msystems.00433-22 PMC9426551

[evad106-B67] Ooka T , et al 2009. Inference of the impact of insertion sequence (IS) elements on bacterial genome diversification through analysis of small-size structural polymorphisms in *Escherichia coli* O157 genomes. Genome Res. 19(10):1809–1816. 19564451 10.1101/gr.089615.108 PMC2765283

[evad106-B68] Pan J , et al 2022. Rates of mutations and transcript errors in the foodborne pathogen *Salmonella enterica* subsp. *enterica*. Mol Biol Evol. 39(4):msac081. 35446958 10.1093/molbev/msac081 PMC9040049

[evad106-B69] Pan J , Williams E, Sung W, Lynch M, Long H. 2021. The insect-killing bacterium *Photorhabdus luminescens* has the lowest mutation rate among bacteria. Mar Life Sci Technol. 3(1):20–27. 33791681 10.1007/s42995-020-00060-0 PMC8009600

[evad106-B70] Pang AW , et al 2010. Towards a comprehensive structural variation map of an individual human genome. Genome Biol. 11(5):R52. 20482838 10.1186/gb-2010-11-5-r52 PMC2898065

[evad106-B71] Parrish N , Sudakov B, Eskin E. 2013. Genome reassembly with high-throughput sequencing data. BMC Genomics. 14 Suppl 1(Suppl 1):S8. 10.1186/1471-2164-14-S1-S8 PMC3549812 23368744

[evad106-B72] Putze J , et al 2009. Genetic structure and distribution of the colibactin genomic island among members of the family Enterobacteriaceae. Infect Immun. 77(11):4696–4703. 19720753 10.1128/IAI.00522-09 PMC2772509

[evad106-B73] Raeside C , et al 2014. Large chromosomal rearrangements during a long-term evolution experiment with *Escherichia coli*. mBio 5(5):e01377-14. 25205090 10.1128/mBio.01377-14 PMC4173774

[evad106-B74] Rausch T , et al 2012. DELLY: structural variant discovery by integrated paired-end and split-read analysis. Bioinformatics 28(18):i333–i339. 22962449 10.1093/bioinformatics/bts378 PMC3436805

[evad106-B75] R Core Team . 2016. R: a language and environment for statistical computing. Vienna, Austria.

[evad106-B76] Sakamoto Y , Zaha S, Suzuki Y, Seki M, Suzuki A. 2021. Application of long-read sequencing to the detection of structural variants in human cancer genomes. Comput Struct Biotechnol J. 19:4207–4216. 34527193 10.1016/j.csbj.2021.07.030 PMC8350331

[evad106-B77] Sawyer SA , et al 1987. Distribution and abundance of insertion sequences among natural isolates of *Escherichia coli*. Genetics 115(1):51–63. 3030884 10.1093/genetics/115.1.51 PMC1203063

[evad106-B78] Schmid M , et al 2018. Pushing the limits of de novo genome assembly for complex prokaryotic genomes harboring very long, near identical repeats. Nucleic Acids Res. 46(17):8953–8965.. 30137508 10.1093/nar/gky726 PMC6158609

[evad106-B79] Schnetz K , Rak B. 1992. IS5: a mobile enhancer of transcription in *Escherichia coli*. Proc Natl Acad Sci U S A. 89(4):1244–1248. 1311089 10.1073/pnas.89.4.1244 PMC48425

[evad106-B80] Sedlazeck FJ , et al 2018. Accurate detection of complex structural variations using single-molecule sequencing. Nat Methods. 15(6):461–468. 29713083 10.1038/s41592-018-0001-7 PMC5990442

[evad106-B81] Sousa A , Bourgard C, Wahl LM, Gordo I. 2013. Rates of transposition in *Escherichia coli*. Biol Lett. 9(6):20130838. 24307531 10.1098/rsbl.2013.0838 PMC3871371

[evad106-B82] Strauch E , Beutin L. 2006. Imprecise excision of insertion element IS 5 from the fliC gene contributes to flagellar diversity in *Escherichia coli*. FEMS Microbiol Lett. 256(2):195–202. 16499606 10.1111/j.1574-6968.2006.00100.x

[evad106-B83] Strauss C , Long H, Patterson CE, Te R, Lynch M. 2017. Genome-wide mutation rate response to pH change in the coral reef pathogen *Vibrio shilonii* AK1. mBio 8(4):e01021-17. 28830944 10.1128/mBio.01021-17 PMC5565966

[evad106-B84] Tham CY , et al 2020. Nanovar: accurate characterization of patients’ genomic structural variants using low-depth nanopore sequencing. Genome Biol. 21(1):56. 32127024 10.1186/s13059-020-01968-7 PMC7055087

[evad106-B85] Thorvaldsdóttir H , Robinson JT, Mesirov JP. 2012. Integrative Genomics Viewer (IGV): high-performance genomics data visualization and exploration. Briefings Bioinf. 14(2):178–192. 10.1093/bib/bbs017 PMC3603213 22517427

[evad106-B86] Tian S , Yan H, Klee EW, Kalmbach M, Slager SL. 2018. Comparative analysis of de novo assemblers for variation discovery in personal genomes. Briefings Bioinf. 19(5):893–904. 10.1093/bib/bbx037 PMC6169673 28407084

[evad106-B87] Tincher C , Long H, Behringer M, Walker N, Lynch M. 2017. The glyphosate-based herbicide roundup does not elevate genome-wide mutagenesis of *Escherichia coli*. G3 7(10):3331–3335. 28983068 10.1534/g3.117.300133 PMC5633383

[evad106-B88] Vandecraen J , Chandler M, Aertsen A, Van Houdt R. 2017. The impact of insertion sequences on bacterial genome plasticity and adaptability. Crit Rev Microbiol. 43(6):709–730.. 28407717 10.1080/1040841X.2017.1303661

[evad106-B89] Van der Auwera GA , et al 2013. From FastQ data to high-confidence variant calls: the Genome Analysis Toolkit best practices pipeline. Curr Protoc Bioinf. 43(1):11.10. 1–11.10. 33. 10.1002/0471250953.bi1110s43 PMC4243306 25431634

[evad106-B90] Wang D , et al 2021. Characterization of gut microbial structural variations as determinants of human bile acid metabolism. Cell Host Microbe. 29(12):1802–1814. e5. 34847370 10.1016/j.chom.2021.11.003

[evad106-B91] Wang X , Wood TK. 2011. IS5 inserts upstream of the master motility operon flhDC in a quasi-Lamarckian way. ISME J. 5(9):1517–1525. 21390082 10.1038/ismej.2011.27 PMC3160685

[evad106-B92] Wick RR . 2019. Badread: simulation of error-prone long reads. J Open Res Softw. 4(36):1316.

[evad106-B93] Wickham H . 2009. Ggplot2: elegant graphics for data analysis. 2nd ed. New York: Springer.

[evad106-B94] Wong KH , Levy-Sakin M, Kwok P-Y. 2018. De novo human genome assemblies reveal spectrum of alternative haplotypes in diverse populations. Nat Commun. 9(1):3040. 30072691 10.1038/s41467-018-05513-w PMC6072799

[evad106-B95] Wu K , et al 2021. Unexpected discovery of hypermutator phenotype sounds the alarm for quality control strains. Genome Biol Evol. 13(8):evab148. 34180992 10.1093/gbe/evab148 PMC8350357

[evad106-B96] Ye K , Schulz MH, Long Q, Apweiler R, Ning Z. 2009. Pindel: a pattern growth approach to detect break points of large deletions and medium sized insertions from paired-end short reads. Bioinformatics 25(21):2865–2871. 19561018 10.1093/bioinformatics/btp394 PMC2781750

[evad106-B97] Zeevi D , et al 2019. Structural variation in the gut microbiome associates with host health. Nature 568(7750):43–48. 30918406 10.1038/s41586-019-1065-y

[evad106-B98] Zhang Z , Schwartz S, Wagner L, Miller W. 2000. A greedy algorithm for aligning DNA sequences. J Comput Biol. 7(1-2):203–214. 10890397 10.1089/10665270050081478

[evad106-B99] Zhao H , et al 2021a. Analysis of 427 genomes reveals moso bamboo population structure and genetic basis of property traits. Nat Commun. 12(1):5466. 34526499 10.1038/s41467-021-25795-x PMC8443721

[evad106-B100] Zhao X , et al 2021b. Expectations and blind spots for structural variation detection from long-read assemblies and short-read genome sequencing technologies. Am J Hum Genet. 108(5):919–928. 33789087 10.1016/j.ajhg.2021.03.014 PMC8206509

