# Supplementary material for: De Novo Structural Variations of Escherichia coli Detected by Nanopore Long-Read Sequencing
Source: Genome Biol Evol. 2023 Jun 9;15(6):evad106. doi: 10.1093/gbe/evad106 (PMC10292909; doi:10.1093/gbe/evad106)
Supplement: evad106_Supplementary_Data [file evad106_supplementary_data.zip › Supplementary File S1-12-23.pdf]

Supplementary File S1

Consistency of the MA datasets with previous ones

From the 67 WT and the 37 Δ*mutS* MA lines, we obtained 270 and 12981 BPSs, respectively (Supplementary Tables S2–S3, S5–S6), yielding mutation rates of 1.95×10^{-10} (95% Poisson Confidence Intervals, 95% CI: 1.72×10^{-10}~2.20×10^{-10}) and 1.76×10^{-8} (95% CI: 1.73×10^{-8}~1.79×10^{-8}) per site per cell division. The mutation rate of Δ*mutS* was about 90 times higher than that of the WT. We also detected 33 and 707 small indels in the WT and the Δ*mutS* MA lines (Supplementary Tables S2–S3, S7–S8), leading to indel mutation rates of 2.38×10^{-11} (95% CI: 1.64×10^{-11}~3.35×10^{-11}) and 9.61×10^{-10} (95% CI: 8.92×10^{-10}~1.03×10^{-9}) per site per cell division. We further calculated the ratio of non-synonymous to synonymous mutations and the ratio of the coding to non-coding mutations (Supplementary Tables S9–S10). The results demonstrate that selection does not bias the mutational features (Lee et al. 2012; Foster et al. 2015; Long et al. 2016). Similar to previous reports, there are more transitions than transversions in the WT and the Δ*mutS* MA lines, especially the latter, which exhibit an extremely high transition to transversion ratio since pre-mutations are prone to transitions (ts/tv for the WT MA lines is 1.78; 44.23 for the Δ*mutS* ones) (Figure S1; Supplementary Table S11); the G:C→A:T transitions occur the most frequently in the WT MA lines, while the A:T→G:C transitions are dominant in the Δ*mutS* lines; the G:C→C:G transversions are the least common type for the two strains (Figure S1; Supplementary Table S11). Nucleotide context does influence BPS mutation rate (Supplementary Figure S2; Supplementary Table S12); there is a deletion bias for the WT MA lines (insertion/deletion ratio of the WT: 0.38, the Δ*mutS*: 0.99), 92.1% of indels of the Δ*mutS* lines occurred in simple sequence repeats (SSR) regions, and 66.7% for the WT lines (Supplementary Tables S6–S7, S11) (Lee et al. 2012; Long et al. 2016). These results are in line with previous studies on the mutation rate of *E. coli* MG1655, especially for the BPS rate, confirming the validity and repeatability of our MA experiments (Supplementary Table S13) (Lee et al. 2012; Foster et al. 2015; Long et al. 2016).

References

Foster PL, Lee H, Popodi E, Townes JP, Tang H. 2015. Determinants of spontaneous mutation in the bacterium *Escherichia coli* as revealed by whole-genome sequencing. *Proc Natl Acad Sci U S A.* 112(44):E5990-E5999. doi: 10.1073/pnas.1512136112.

Lee H, Popodi E, Tang H, Foster PL. 2012. Rate and molecular spectrum of spontaneous mutations in the bacterium *Escherichia coli* as determined by whole-genome sequencing. *Proc Natl Acad Sci U S A.* 109(41):E2774-E2783. doi: 10.1073/pnas.1210309109.

Long H, et al. 2016. Antibiotic treatment enhances the genome-wide mutation rate of target cells. *Proc Natl Acad Sci U S A.* 113(18):E2498-E2505. doi: 10.1073/pnas.1601208113.
