# Supplementary material for: De Novo Structural Variations of Escherichia coli Detected by Nanopore Long-Read Sequencing
Source: Genome Biol Evol. 2023 Jun 9;15(6):evad106. doi: 10.1093/gbe/evad106 (PMC10292909; doi:10.1093/gbe/evad106)
Supplement: evad106_Supplementary_Data [file evad106_supplementary_data.zip › Supplementary Figures-12-23.pdf]

Supplementary Figures

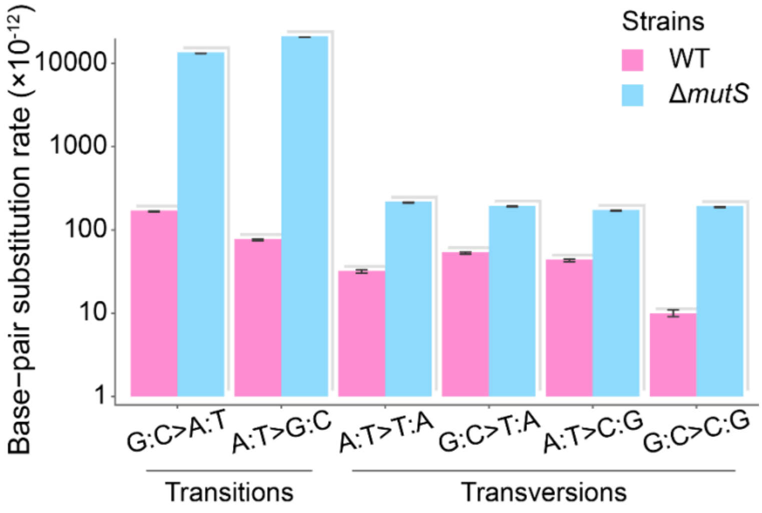

Supplementary Figure S1. Mutation spectra of the wild-type (WT) and Δ*mutS* MA lines. The G:C>A:T represents G→A plus C→T transition mutations, and similar for other labels. The base-pair substitutions rate is in the units of per site per cell division.

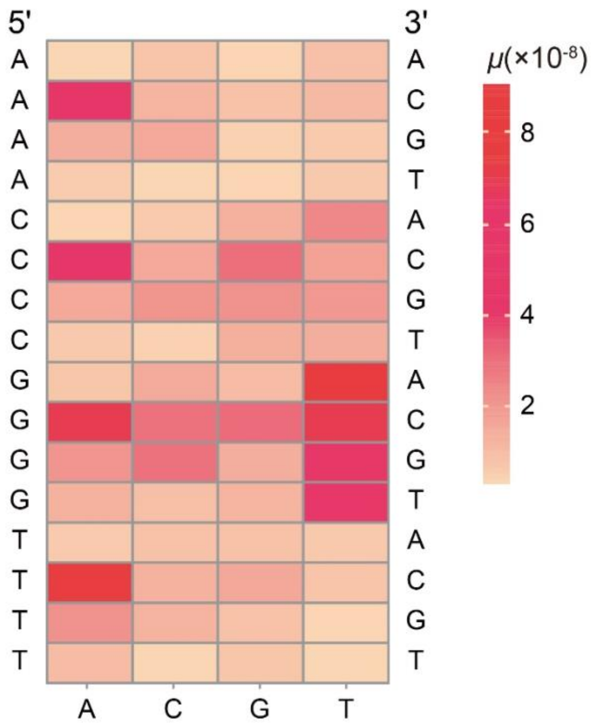

Supplementary Figure S2. The context-dependent BPS mutation rates of Δ*mutS* MA lines. Each colored block represents the mutation rate of one nucleotide with certain neighboring nucleotide context. The upstream (5'-flanking) nucleotide are marked on the left, and the downstream nucleotide are marked on the right, and focal bases are shown at the bottom. μ is BPS rate in the unit of per site per cell division, and is calculated by number of mutations with the context divided by the product of the total number of the tri-nucleotide context in the genome × the MA line number × the mean number of cell divisions.

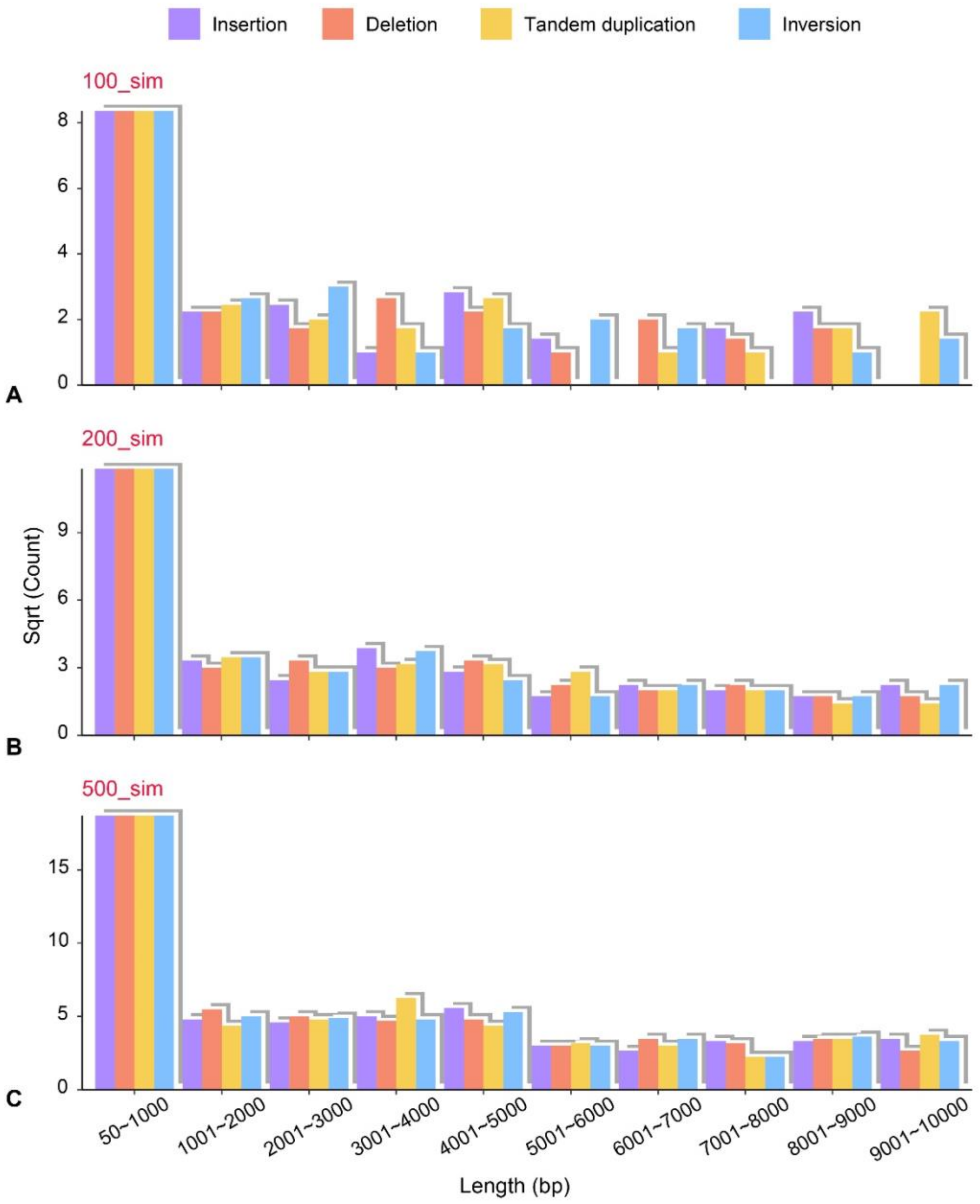

Supplementary Figure S3. Statistics of the length distribution of the SVs in the four groups of simulated genomes (insertion, deletion, tandem duplication and inversion). Simulated genomes with 100 SVs (**A**), 200 SVs (**B**), 500 SVs (**C**) in the four groups. The y-axis is the square root of the number of SVs.
